# MicroRNA‐130a controls bone marrow mesenchymal stem cell differentiation towards the osteoblastic and adipogenic fate

**DOI:** 10.1111/cpr.12688

**Published:** 2019-09-26

**Authors:** Zhangyuan Lin, Hongbo He, Min Wang, Jieyu Liang

**Affiliations:** ^1^ Department of Orthopedic Xiangya Hospital of Central South University Changsha China; ^2^ Department of Endocrinology Xiangya Hospital of Central South University Changsha China

**Keywords:** adipogenesis, age, cell differentiation, osteogenesis, osteoporosis

## Abstract

**Objectives:**

With age, bone marrow mesenchymal stem cells (BMSC) have reduced ability of differentiating into osteoblasts but have increased ability of differentiating into adipocytes which leads to age‐related bone loss. MicroRNAs (miRNAs) play major roles in regulating BMSC differentiation. This paper explored the role of miRNAs in regulating BMSC differentiation swift fate in age‐related osteoporosis.

**Material and methods:**

Mice and human BMSC were isolated from bone marrow, whose miR‐130a level was measured. The abilities of BMSC differentiate into osteoblast or fat cell under the transfected with agomiR‐130a or antagomiR‐130a were analysed by the level of ALP, osteocalcin, Runx2, osterix or peroxisome proliferator‐activated receptorγ (PPARγ), Fabp4. Related mechanism was verified via qT‐PCR, Western blotting (WB) and siRNA transfection. Animal phenotype intravenous injection with agomiR‐130a or agomiR‐NC was explored by Micro‐CT, immunochemistry and calcein double‐labelling.

**Results:**

MiR‐130a was dramatically decreased in BMSC of advanced subjects. Overexpression of miR‐130a increased osteogenic differentiation of BMSC and attenuated adipogenic differentiation in BMSC, conversely, Inhibition of miR‐130a reduced osteogenic differentiation and facilitated lipid droplet formation. Consistently, overexpression of miR‐130a in elderly mice dropped off the bone loss. Furthermore, the protein levels of Smad regulatory factors 2 (Smurf2) and PPARγ were regulated by miR‐130a with an negative effect through directly combining the 3'UTR of Smurf2 and PPARγ.

**Conclusions:**

The results indicated that miR‐130a promotes osteoblastic differentiation of BMSC by negatively regulating Smurf2 expression and suppresses adipogenic differentiation of BMSC by targeting the PPARγ, and supply a new target for clinical therapy of age‐related bone loss.

## 1 INTRODUCTION

Age‐related bone loss made the risk of fractures among elderly individuals raised, which is believed to occur due to lessened bone formation and enhanced marrow fat accumulation.^1^, ^2^, ^3^ BMSC have the ability of differentiating into variety of cells, including osteoblasts and adipocytes.^4^, ^5^, ^6^, ^7^ It has been reported, during ageing, BMSC have reduced ability of differentiating into osteoblasts but have increased ability of differentiating into adipocytes which causes age‐related bone loss.^8^, ^9^, ^10^ However, this mechanism still needs to further investigate.

MicroRNAs (miRNAs), which means small non‐coding RNAs, always act as an negative regulate factor over the process of expression of target genes by degrading mRNAs or inhibiting the translation of mRNAs.^11^, ^12^, ^13^ Recently, several studies have proved the importance of miRNAs in regulating osteoblastic differentiation and adipogenic differentiation.^14^, ^15^, ^16^, ^17^ However, roles of miRNAs in BMSC differentiation shift fate from osteoblasts to adipocytes during ageing are still unclear.

In this paper, we explored the action of miR‐130a in BMSC differentiation during ageing as well as mechanisms during the process. We demonstrated that miR‐130a was significantly decreased in aged mice and human subjects. MiR‐130a promotes osteoblastic differentiation of BMSC through regulating Smurf2 expression and inhibits adipogenic differentiation of BMSC by targeting the PPARγ at the post‐transcriptional level. Furthermore, inhibition of miR‐130a in young mice develops a low bone mass. Overexpression of miR‐130a in elderly mice dropped off the bone loss. Therefore, our conclusion supplied a novel mechanism and target for clinical therapy of age‐related bone loss.

## 2 METHODS

### 2.1 Mice

We separated 15‐month‐old mice into two groups with intravenous injection of agomiR‐130a or agomiR‐NC, respectively, twice per week for 3 months. Animal care and experiment were all approved by the Institutional Animal Care and Use Committee of the Laboratory Animal Research Center in Xiangya Medical School of Central South University.

### 2.2 BMSC isolation and culture

We isolated and collected BMSC of mouse as before, as well as the cultivation.^18^ In order to isolate BMSC from medullary cavity, female mice were killed, and BMSC were washed out from femora and incubated at 4°C for 20 minutes with FITC‐, PE‐ and allophycocyanin‐conjugated antibodies, and peridinin chlorophyll‐protein that combined with CD29, CD45, CD11b and Sca‐1 (BioLegend). For the isolation of human BMSC, we use the same method to harvest human BMSC. The human BMSC were incubated at 4°C for 30 minutes with antibody of allophycocyanin‐, FITC‐ and PE‐conjugated which recognized CD45, CD146 and STRO‐1 (BioLegend). By using FACS Aria model and FACS DIVE software version 6.1.3(BD Biosciences), acquisition was carried out and the analysis was enforced.

Here, we find out that the mouse BMSC were sorted as CD29+Sca‐1+CD45−CD11b‐, while human BMSC (hBMSC) were sorted as CD146+STRO‐1+CD45‐. Then, we gathered and cultured them for 1‐2 weeks. In culture flasks, the primary BMSC were separated and seeded for cell population enrichment. Approximately 1 week later, as the second‐passage BMSC reached clustered, they were subcultured. Afterwards, we induced adipogenic and osteogenic differentiation in the third‐passage BMSC. Plasmid transfection also executed in third‐passage BMSC.

### 2.3 Histochemistry analysis

The histochemistry analysis was conducted as documented before.^19^, ^20^ In short, after euthanasia, we gathered mouse femora and fixed them in 10% formalin for 1 day. After performing that, we transfer mouse femora in 10% EDTA for 2 weeks. Finally, we embedded the bone with paraffin decalcified. H&E, toluidine blue and TRAP staining were performed in 4‐μm bone sections to calculate number and surface of osteoblasts and osteoclasts, as well as adipocytes. To measure the histomorphometry of 2‐dimensional parameters of bones, the OsteoMeasureXP Software (OsteoMetrics Inc) was in use. At 8 and 2 days before euthanasia, mice were intraperitoneally injected with 25 mg/kg calcein. We fixed the mouse femora in 70% ethanol, then dehydrated it with increasing concentration gradient of ethanol, finally embedded it with methyl methacrylate. The femur was sliced into serial 5‐μm sections with using a microtome. For quantitative estimate the situation of bone formation, the parameters like number of osteoblast, bone formation rate and osteoblast surface were obtained. The parameters of osteoclast number and osteoclast surface which represented bone resorption were acquired also.

### 2.4 Immunohistochemical staining

As previously described, immunohistochemical staining was performed.^21^, ^22^ In short, for antigen retrieval, bone sections were performed for 15 minutes by digestion with 0.05% trypsin. After that, the bone sections were incubated with primary antibody which against osteocalcin (Takara) at 4°C overnight. Later, we performed counterstaining with haematoxylin (Sigma‐Aldrich) to detect the immunoactivity. HRP‐streptavidin detection system (Dako) was made use of. As negative controls, polyclonal rabbit IgG (R&D Systems Inc) was used to incubated with sections.

### 2.5 Calcein double‐labelling

Calcein double‐labelling was performed as we demonstrated before.^23^ Mice were intraperitoneally injected with 0.08% calcein (Sigma‐Aldrich, 20 mg/kg b.w.) at 8 and 2 days before animal euthanasia executed. We observed bone slices under a fluorescence microscope with decalcified. We set the line of 8 days before euthanasia as a baseline for calcein double‐labelling analysis and compare the length between two lines. We selected four visual regions at random from the distal metaphysis of femur and took measurement.

To analyse the action of miR‐130a, PCR amplify was performed in the 3′‐UTR segments of PPARγ and Smurf2, which include the miR‐130a‐binding site we have predicted before. Both humans and mouse were inserted refined PCR products instantly downstream of the stop codon of the pGL3 control luciferase reporter vector (Promega Corp.) in XbaI‐FseI site. In this step, we created the WT‐pGL3‐PPARγ and WT‐pGL3‐Smurf2. Then, we took advantage of QuikChange Site‐Directed Mutagenesis Kit (Stratagene) for the preparation of PPARγ and Smurf2 mutants. So that the miR‐130a seed regions could get MUT‐pGL3‐PPARγ and MUT‐pGL3‐Smurf2 in humans and mouse. The agomiR‐NC or agomiR‐130a and pRL‐TK renilla luciferase plasmid (Promega Corp.) were applied for 2 days with Lipofectamine 2000 (Invitrogen). Either mutant pGL3 or WT construct was transfected into human and mouse BMSC. After that, to quantify luminescent signal, luminometer (Glomax; Promega Corp.) and the dual‐luciferase reporter assay system (Promega Corp.) were utilized.

Finally, we use cotransfected phRL‐null vector (Promega Corp.) to normalize the renilla luciferase value in the firefly luciferase assay.

### 2.6 Assay of adipogenic differentiation

We induced BMSC adipogenic differentiation in vitro as before.^24^ BMSC were cultured with adipogenesis induction medium (α‐MEM containing 10% FCS, 5 μg/mL insulin, 0.5 mmol/L 3‐isobutyl‐1‐methylxanthine, and 1 μmol/L dexamethasone) in 6‐well plates with density of 2.5 × 10^6^ cells per well for 14 days. Every other day, we changed culture medium. We did Oil Red O staining to distinguish mature adipocytes from preadipocyte during the process of culture.

### 2.7 Osteogenic differentiation and mineralization assay

Osteoblasts were collected as before.^25^ BMSC were cultured using twenty‐four‐well plates (5 × 105 cells/well) for 48 hours by applying the osteogenesis induction medium (5 mmol/L β‐glycerophosphate and 50 μg/mL ascorbic acid, 300 ng/mL BMP‐2). Then, homogenize the cell lysates to make the evaluation of ALP activity by utilizing the enzymatic colorimetric ALP Kit (from Roche) through spectrophotometric measurement of p‐nitrophenol's output. Secreted osteocalcin levels were made an assessment in culture media by applying an immunoassay kit (DiaSorin).

Applying medium can induce osteogenesis to culture BMSC in six‐well plates with a concentration of 2.5 × 106 cells per well for twenty‐one days to make osteoblastic mineralization induction. After that, two per cent of Alizarin Red‐S (Sigma‐Aldrich) was utilized for staining cells at PH 4.2 in order to make an evaluation of the mineralization of the cell matrix. Camera System (Nikon), as well as a Diaphot Inverted Microscope, was utilized for further imaging. Make the quantification of the concentration of Alizarin Red‐S through spectrophotometry at 540 nm, which was discharged from the cell matrix to the cetylpyridinium chloride solution.

Under the use of the Bradford assay, a portion of lysate solution was subjected for the purpose of normalizing protein expression into complete cellular protein.

### 2.8 qRT‐PCR

In previous description, Roche Molecular Light Cycler was applied for qRT‐PCR.^26^, ^27^ TRIzol reagent (Invitrogen) was utilized for isolating RNA originated from tissues/cultured cells. We apply one microgram total RNA as well as SuperScript II (Invitrogen) to carry out reverse transcription. Then, performing amplification reactions by using amplification primers with SYBR Green PCR Master Mix (PE Applied Biosystems), whose reaction volumes were 25‐microlitre request. The one microlitre volume of cDNA was applied in every set of experiments.

### 2.9 Western blot

As was described in the previous study,^26^ Western blot was performed with the following steps. Total cell lysate isolation was made with SDS‐PAGE, which followed with blotting on PVDF membranes (Millipore). After incubation with Smurf2, PPARγ or β‐actin (Abcam) antibodies, the membranes were re‐probed with suitable horseradish peroxidase‐conjugated secondary antibodies. Application of an ECL kit (Santa Cruz) assists the maintenance of blots; then, an X‐ray film exposure was applied.

### 2.10 Microcomputed tomography analysis

The micro‐CT analysis was performed as previously described.^28^ Make dissection of mouse right femur and lumbar, and fix these specimens in 4% PFA for 24 hours. We use a GE Explore Locus SP microcomputed tomography (μCT) system (GE Healthcare Co.) to scan and analyse specimens. 80 kV voltage and 80 μA current of X‐ray were utilized and maintained during scanning procession with 12 μm per pixel resolution. Trabecular bone 3D histomorphometric analysis was performed with cross‐sectional images of the L4 vertebra and distal femur application. We obtained and analysed our region of interest (ROI) data, which include five per cent of the femoral length from 100 µm under the growth plate in the distal femur. The results contain trabecular thickness (Tb. Th), trabecular separation (Tb. Sp), trabecular number (Tb. N) and trabecular bone volume per tissue volume (Tb. BV/TV). We select the total area from a trabecular bone in the L4 vertebra to make the analysis of the vertebral trabecular bone volume per tissue volume (Vt. BV/TV). The midshaft of the femur cross‐sectional images, whose ROI was determined as ten per cent of femoral length in the middle of the femur, was utilized for 3D histomorphometric analysis. The results include periosteal cortical thickness (Ct. Th), perimeter (Ps. Pm) and endosteal perimeter (Es. Pm).

### 2.11 The three‐point bending test

The three‐point bending test was performed followed the previous study.^29^ A mechanical‐testing machine (WDW3100; Changchun, China), which was equipped with a 500 NM‐SI sensor (Celtron Technologies Inc), was applied to make a measurement at the midshaft location of the tibia and femur. The three‐point test was made up of two support points in the end and one loading point locate in the central. The length spans between the two end‐support points account for sixty per cent of the entire length of the bone. Loading each bone specimen at a speed of 0.155 mm per second until failure. Obtaining the bio‐mechanical measurement data from the load‐deformation curves accompany with the records of the maximum load (Newton) and stiffness (Newton per millimetre).

### 2.12 Population study

All participants’ written informed consent was obtained before collecting bone marrow. The participants consist of 22 female and 26 male, and their age ranged from 20 to 79 years old and received hip or joint replacement treatment. All clinical bone marrow specimens were served by the Ethics Committee of the Xiangya Hospital of Central South University. This study meets recognized standards.

### 2.13 Statistics

Statistics analysis shows mean ± SD. We applied 2‐tailed Student's *t* test to analyse and compare two groups. One‐way ANOVA was performed to make comparisons among multiple groups. Three times repeating experiments are required, and symbolic experiments are presented before. The significant difference was identified when *P* < .05.

## 3 RESULT

### 3.1 MiR‐130a decreased in BMSC during ageing process

**Figure 1:**
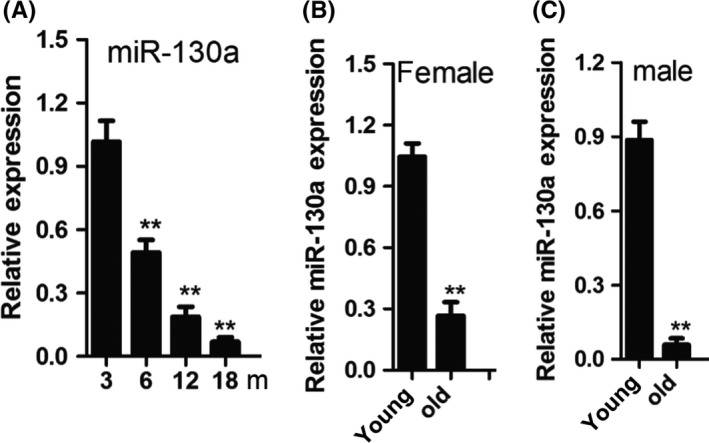
MiR‐130a was obviously decreased in BMSC during ageing. A, qRT‐PCR analysis of the levels of miR‐130a expression in BMSC derived from the mice at different ages. n = 6 per group. B and C, Age‐associated changes in miR‐130a levels in BMSC from 22 human females B, and 26 males C. Data shown as mean ± SD. **P < .01 (A, ANOVA; B and C, Student's *t* test)

The conclusion that MiR‐130a level in BMSC was prominent lower in aged mice (18 months) than young mice (3 months) using miRNA microarray technology was demonstrated in previous research. In order to ascertain the expression of miR‐130a in the mice with different ages, we isolated Sca‐1+CD29+CD45−CD11b‐ mesenchymal stem cells from bone marrow cells 30 in mice at 3, 6, 12 and 18 months and quantitative real‐time RT‐PCR (qRT‐PCR) was implemented. We can draw a conclusion that the miR‐130a expression declined corresponding to the progress of ageing (Figure 1A). Then, we gathered human BMSC (defined as STRO‐1+CD146+CD45‐) 31 from bone marrow cells of femora in both young and aged specimens through FACS. miR‐130a expression levels were significantly decreased in elder group than the younger group (Figure 1B and C). The results indicate that miR‐130a may influence the differentiation orientation of BMSC in the process of ageing.

### 3.2 MiR‐130a promotes osteoblastic differentiation of BMSC

**Figure 2:**
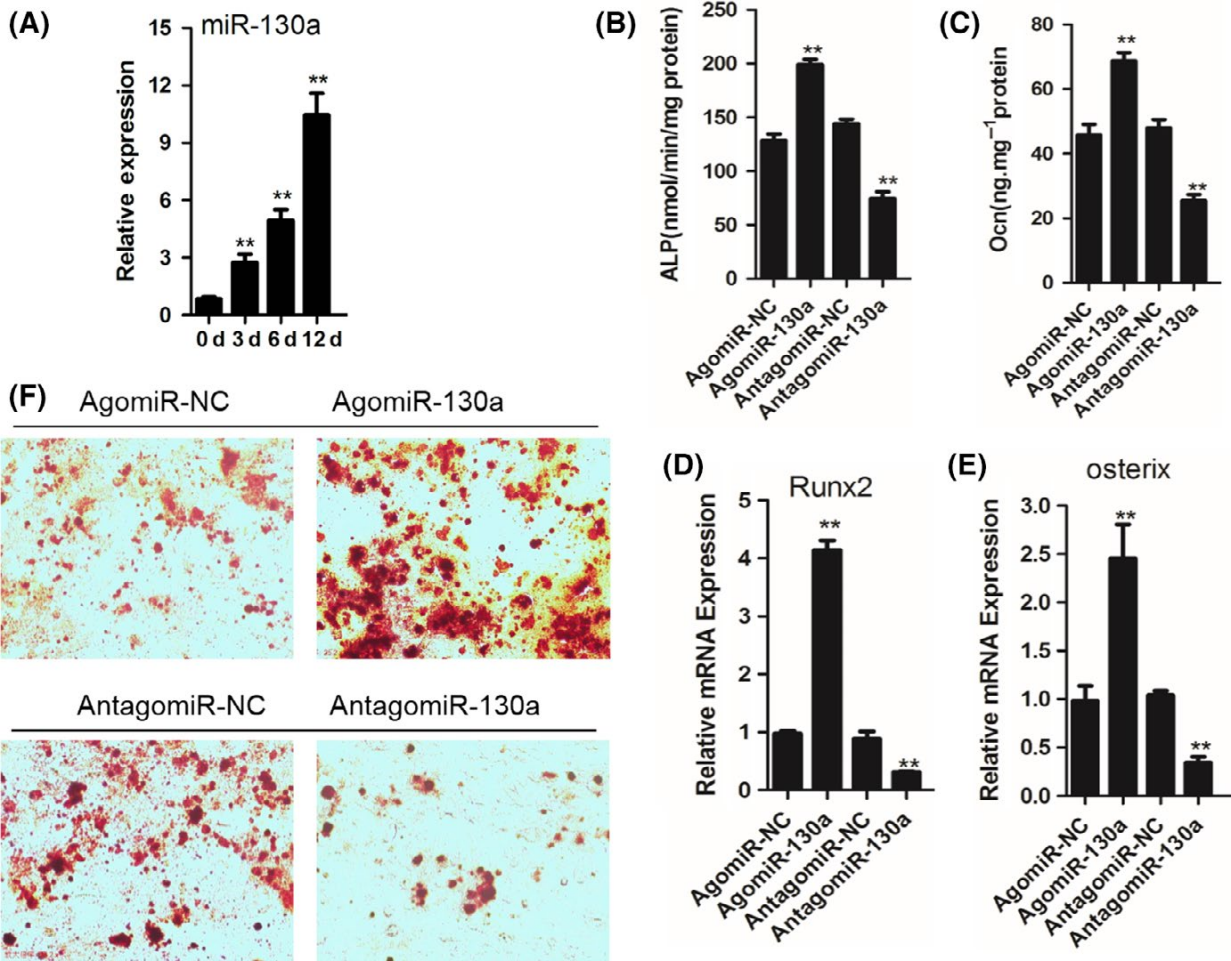
MiR‐130a promotes osteoblastic differentiation of BMSC. A, qRT‐PCR analysis of the relative levels of miR‐130a expression in BMSC cultured in osteogenesis induction medium (300 ng/mL BMP‐2, 50 μg/mL ascorbic acid and 5 mmol/L β‐glycerophosphate) for the days as indicated. n = 5 per group. B and C, Analysis of ALP activity B, and osteocalcin secretion C, in BMSC transfected with agomiR‐130a, antagomiR‐130a or their controls cultured in osteogenesis induction medium for 48 hours. D and E, qRT‐PCR analysis of the relative levels of Runx2 D, and osterix E, mRNA expression in BMSC cultured in osteogenesis induction medium for 48 hours. n = 5 per group. F, Representative images of Alizarin Red‐S staining of BMSC cultured in osteogenesis induction medium for 48 hours. Data shown as mean ± SD. **P < .01 (ANOVA)

Measured by qRT‐PCR (Figure 2A), MiR‐130a expression gradually increased during osteoblastic differentiation in mouse BMSC. To clarify the effect of miR‐130a on osteoblastic differentiation, agomiR‐130a or antagomiR‐130a was transfected in BMSC to overexpress or suppress miR‐130a, respectively. Osteocalcin and alkaline phosphatase (ALP) were utilized as osteoblast differentiation markers. In our study, after induction of osteoblastic differentiation for 48 hours, osteocalcin secretion and ALP activity were higher in the group of agomiR‐130a–transfected cells than the control group. By contrast, these two markers were down‐regulated in antagomiR‐130a‐transfected cells (Figure 2B and C). Also, RT‐PCR results reveal that the mRNA expression levels of Runx2 and osterix were obviously enhanced in agomiR‐130a transfection group. In contrast, antagomiR‐130a transfection inhibited Runx2 and osterix expression (Figure 2D and E). Furthermore, the differentiation of osteogenic BMSC was gauged by Alizarin Red staining after induction of osteoblastic differentiation for 21 days. Alizarin Red staining indicates increased mineralized nodule formation in BMSC with agomiR‐130a transfection and decreased mineralized nodule formation in BMSC with antagomiR‐130a transfection (Figure 2F). All these data implied that miR‐130a accelerates osteoblastic differentiation of BMSC.

### 3.3 MiR‐130a inhibits adipogenic differentiation of BMSC

**Figure 3:**
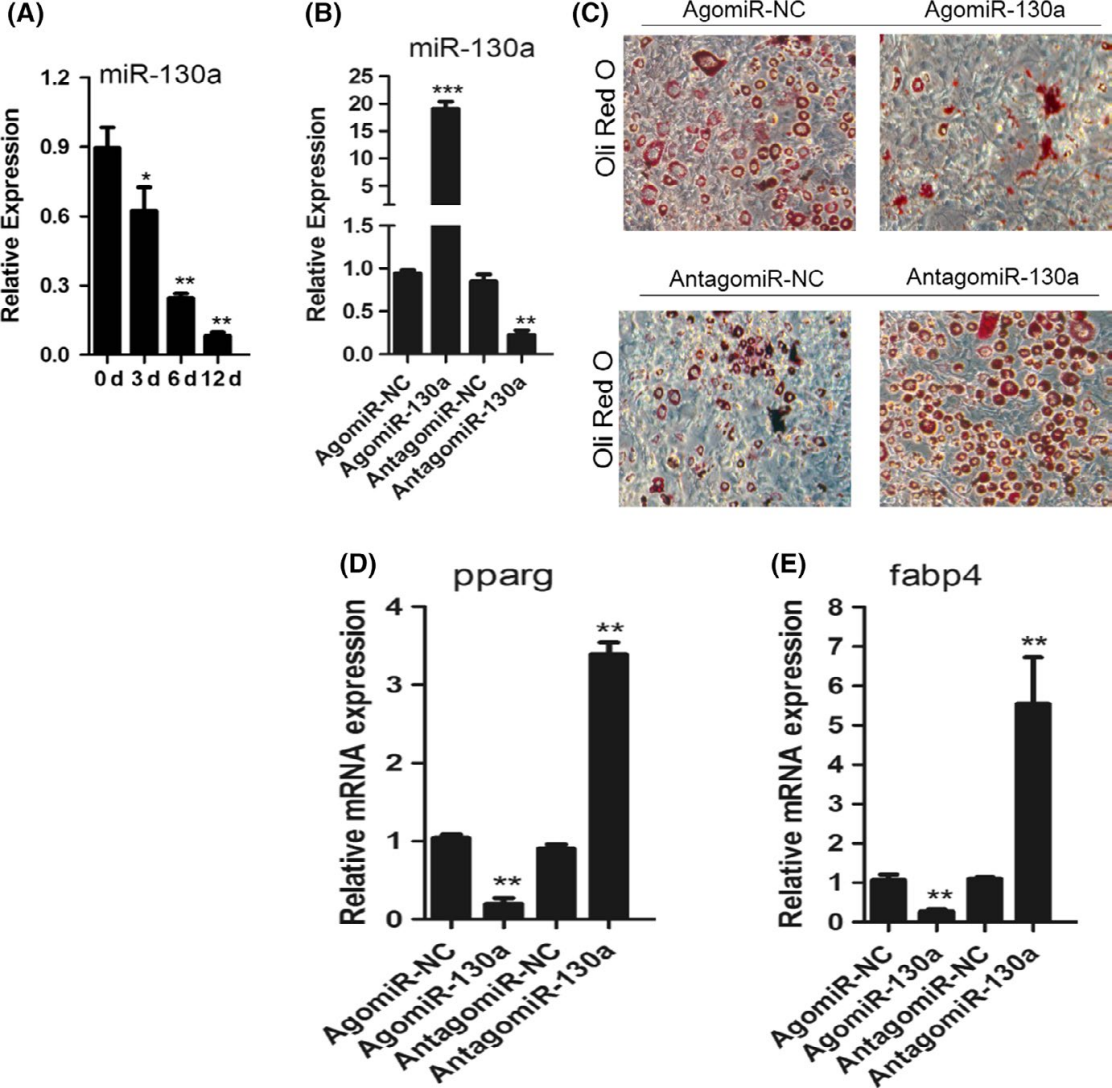
MiR‐130a inhibits adipogenic differentiation of BMSC. A, qRT‐PCR analysis of the relative levels of miR‐130a expression in BMSC cultured in adipogenesis induction medium (0.5 mmol/L 3‐isobutyl‐1‐methylxanthine, 5 μg/mL insulin and 1 μmol/L dexamethasone) for the days as indicated. n = 5 per group. B, qRT‐PCR analysis of the relative levels of miR‐130a expression in BMSC transfected with 10 μmol/L agomiR‐130a, antagomiR‐130a or their negative controls. NC, negative control. n = 5 per group. C, Representative images of Oil Red O staining of lipids in BMSC cultured in adipogenesis induction medium for 14 days. Scale bar: 100 μm. D and E, qRT‐PCR analysis of the relative levels of PPARG D, and Fabp4 E, mRNA expression in BMSC cultured in adipogenesis induction medium for 48 hours. Data shown as mean ± SD. *P < .05, **P < .01, ***P < .001 (ANOVA)

BMSC culture needs an adipogenesis induction medium. The reduced expression of MIR‐130a is obvious during adipogenic differentiation of mouse BMSC (Figure 3A).

AgomiR‐130a or antagomiR‐130a was utilized to transfect BMSC and have them overexpress or silence miR‐130a (Figure 3B). Overexpression of miR‐130a strengthened the formation of lipid droplets, while adipogenic differentiation of mouse BMSC was weakened while silencing miR‐130a (Figure 3C). Meanwhile, the mRNA levels of PPARγ and fatty acid binding protein 4 (Fabp4), which were the important markers of adipocyte differentiation, were inhibited due to the overexpression of miR‐130a in BMSC. On the contrary, the mRNA expression of PPARγ and Fabp4 was elevated accompany with silencing of miR‐130a (Figure 3D and E). Therefore, miR‐130a inhibits adipogenic differentiation of BMSC.

### 3.4 Overexpression of miR‐130a induces bone loss reduction in aged mice

**Figure 4:**
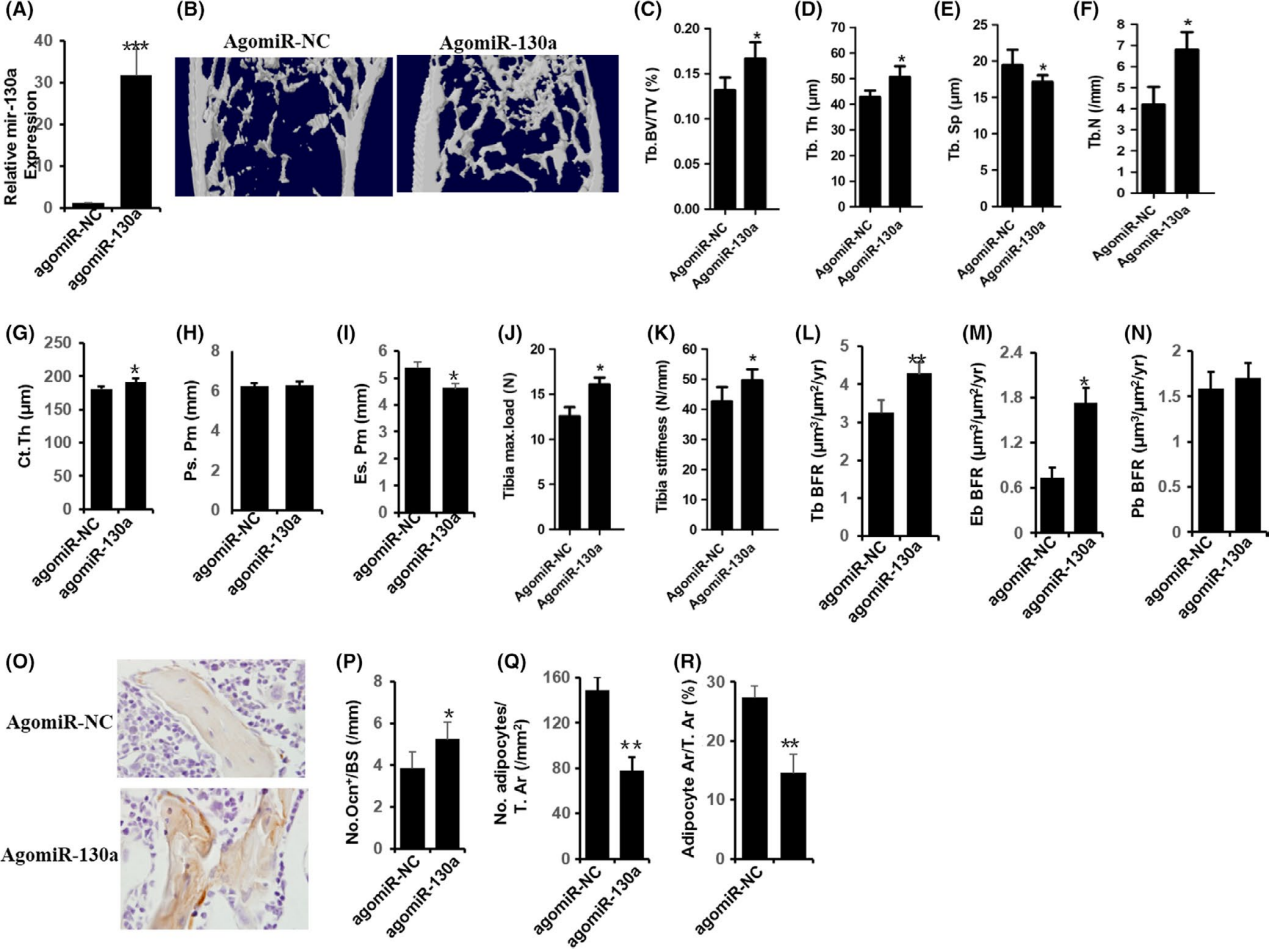
Overexpression of miR‐130a reduces bone loss in aged mice. A, qRT‐PCR analysis of levels of miR‐130a expression in BMSC of mice with agomiR‐130a or agomiR‐NC. NC, negative control. n = 6 per group. B‐I, Representative μCT images B, and quantitative μCT analysis of trabecular C‐F, and cortical bone G‐I, microarchitecture in femora of mice with agomiR‐NC or agomiR‐130a. n = 10. J and K, Three‐point bending measurement of femur maximum load J and stickness K. n = 5 per group. L‐N, Calcein double‐labelling–based quantification of bone formation rate per bone surface (BFR/BS) in femora. n = 5 per group. O‐R, Representative images of osteocalcin immunohistochemical staining O, and quantification of number of osteoblasts P, number and area of adipocytes Q and R, and in distal femora. Scale bars: 100 μm. n = 5 per group. Data shown as mean ± SD. *P < .05, ***P < .001 (Student's *t* test)

Intravenously injection with agomiR‐130a or agomiR‐NC twice per week for three months was applied on mice which were 15 months old. According to a qRT‐PCR outcome, it shows that the expression of miR‐130a in bone tissue rose apparently caused by agomiR‐130a injection (Figure 4A). Comparing with vehicle‐treated mice, the mice treated with agomiR‐130a presented with significantly higher cortical bone thickness and trabecular bone volume per tissue volume, lower trabecular bone separation and trabecular bone number (Figure 4B‐I). While, agomiR‐130a–treated mice showed greater bone strength, which presented with the higher outcome of the tibia maximum load as well as bone stiffness than the vehicle‐treated group (Figure 4J and K). In addition, agomiR‐130a–treated group showed obviously increased bone formation rate which demonstrated with the significantly higher value of endosteal and trabecular bone formation rates (BFRs) than mice treated with agomiR‐NC (Figure 4L‐N). Furthermore, noticeable higher osteoblast number and occupied surface area, and the number, as well as the area of adipocytes occupied on the endosteal and trabecular bone surfaces in the bone marrow, were distinctly lower in agomiR‐130a–treated mice (Figure 4O‐R) with a comparison to vehicle‐treated mice. These results indicate the accumulation of bone marrow fat is suppressed and the formation of bone is promoted in mice overexpressing miR‐130a.

### 3.5 MiR‐130a targets the 3′UTR of Smurf2 mRNA to regulate osteogenic differentiation

**Figure 5:**
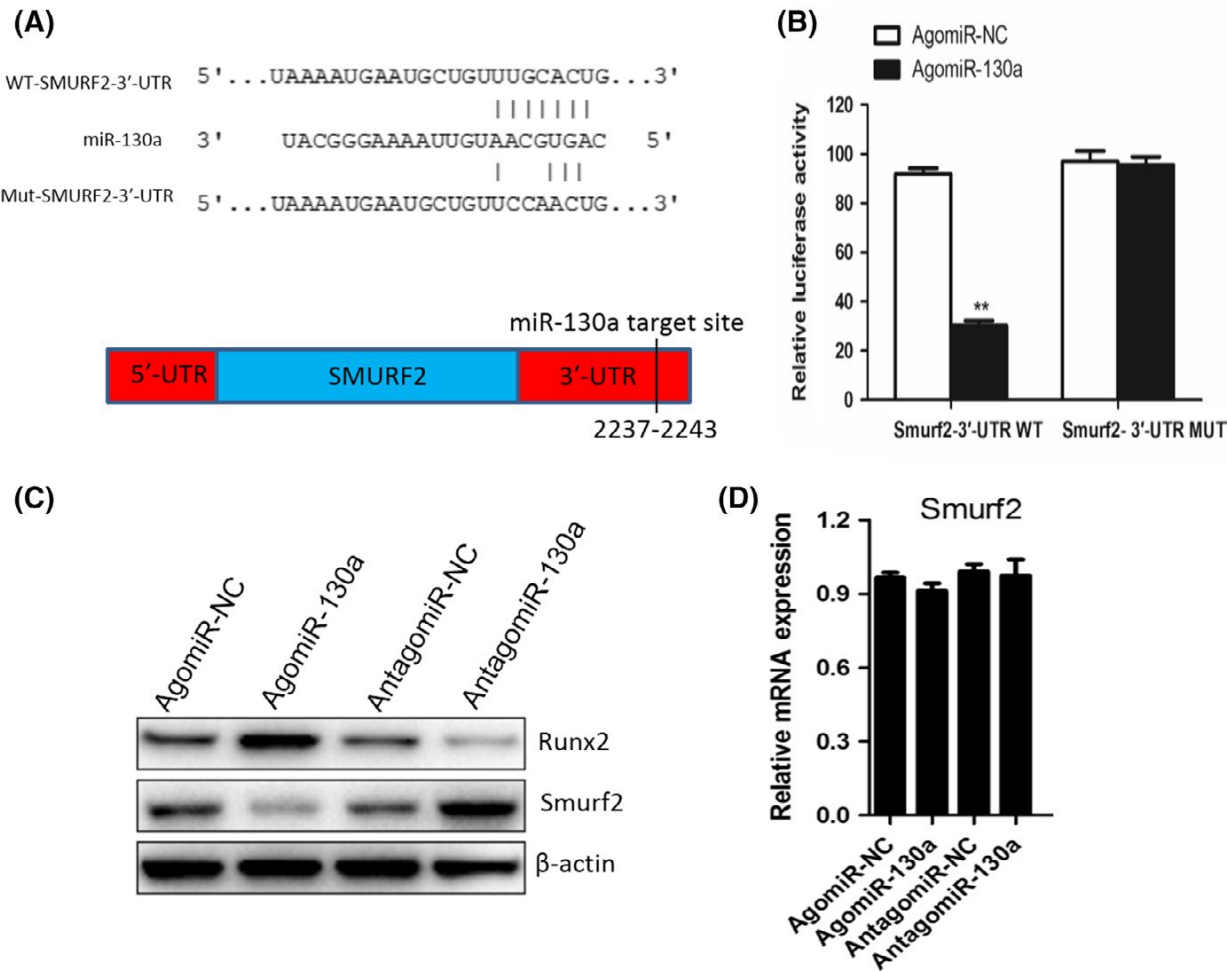
MiR‐130a directly targets the 3′UTR of Smurf2 mRNA to regulate osteogenic differentiation. A, Schematic of miR‐130a putative target sites in mouse SMURF2 3′‐UTR. CDS, coding sequence. B, BMSC were transfected with luciferase reporter carrying WT or MUT 3′‐UTR of the SMURF2 gene (SMURF2‐3′‐UTR WT and SMURF2‐3′‐UTR MUT). Effects of miR‐130a on the reporter constructs were determined at 48 hours after transfection. Firefly luciferase values, normalized for renilla luciferase, are presented. n = 3 per group. C, Western blot analysis of the relative levels of Runx2 and Smurf2 protein expression in BMSC transfected with agomiR‐130a and antagomiR‐130a. β‐Actin was used as loading control. Data are representative of 3 independent experiments. D, qRT‐PCR analysis of levels of Smurf2 mRNA expression in BMSC transfected with agomiR‐130a and antagomiR‐130a. n = 3 per group. Data shown as mean ± SD. **P < .01 vs. agomiR‐NC or agomiR‐130a (Student's *t* test)

It has been accepted that miRNA binds to complementary sequences of target mRNAs which located in 3′‐UTR, so the mRNA expression of target genes can be inhibited.^32^, ^33^, ^34^ Starbase v2.0 was the tool used for predicting the possible target genes of miR‐130a,^35^ PicTar^36^ and TargetScan,^37^ and medium stringency was used. Of all the genes that has been predicted potential targets in both databases, we chose Smurf2, which is an important transcription factors inhibiting osteogenesis.^38^ By sequence analysis, MiR‐130a had potential binding sites in the 3'UTR of Smurf2 in human and rat (Figure 5A). To identify whether miR‐130a can directly target Smurf2 3'UTR, luciferase report vectors were generated with wild‐type Smurf2 3'UTR (pGL3‐smurf2‐WT) and mutated Smurf2 3'UTR (pGL3‐Smurf2‐MUT). The Smurf2 luciferase expression vector and activity were measured for describing miR‐130a function on luciferase translation. Luciferase activity of Smurf2 was inhibited by miR‐130a significantly, yet pGL3‐Smurf2‐MUT relieved this effect (Figure 5B). Then, we proved that Smurf2 is the direct target of miR‐130a.

Moreover, we measured the Smurf2 and Runx2 protein expression levels. As expected, agomiR‐130a down‐regulated Smurf2 protein expression while antagomiR‐130a up‐regulated its expression (Figure 5C); however, Smurf2 mRNA level did not alter (Figure 5D). Then, we got the conclusion that miR‐130a targets Smurf2 and regulates Smurf2 expression in post‐transcription.

It is believed that Runx2 is a specific osteoblast transcription factor, which plays essential roles in bone formation.^39^, ^40^ agomiR‐130a up‐regulated Runx2 expression while antagomiR‐130a down‐regulated it (Figure 5C). It is well known that Smurf2 can mediate suppression of Runx2 transcriptional activity.^41^ Hence, based on our results, we suggest that miR‐130a promotes osteoblast differentiation by alleviating the Smurf2‐mediated suppression of Runx2 transcriptional activity.

### 3.6 MiR‐130a targets the 3′UTR on PPARγ mRNA to regulate adipogenic differentiation

**Figure 6:**
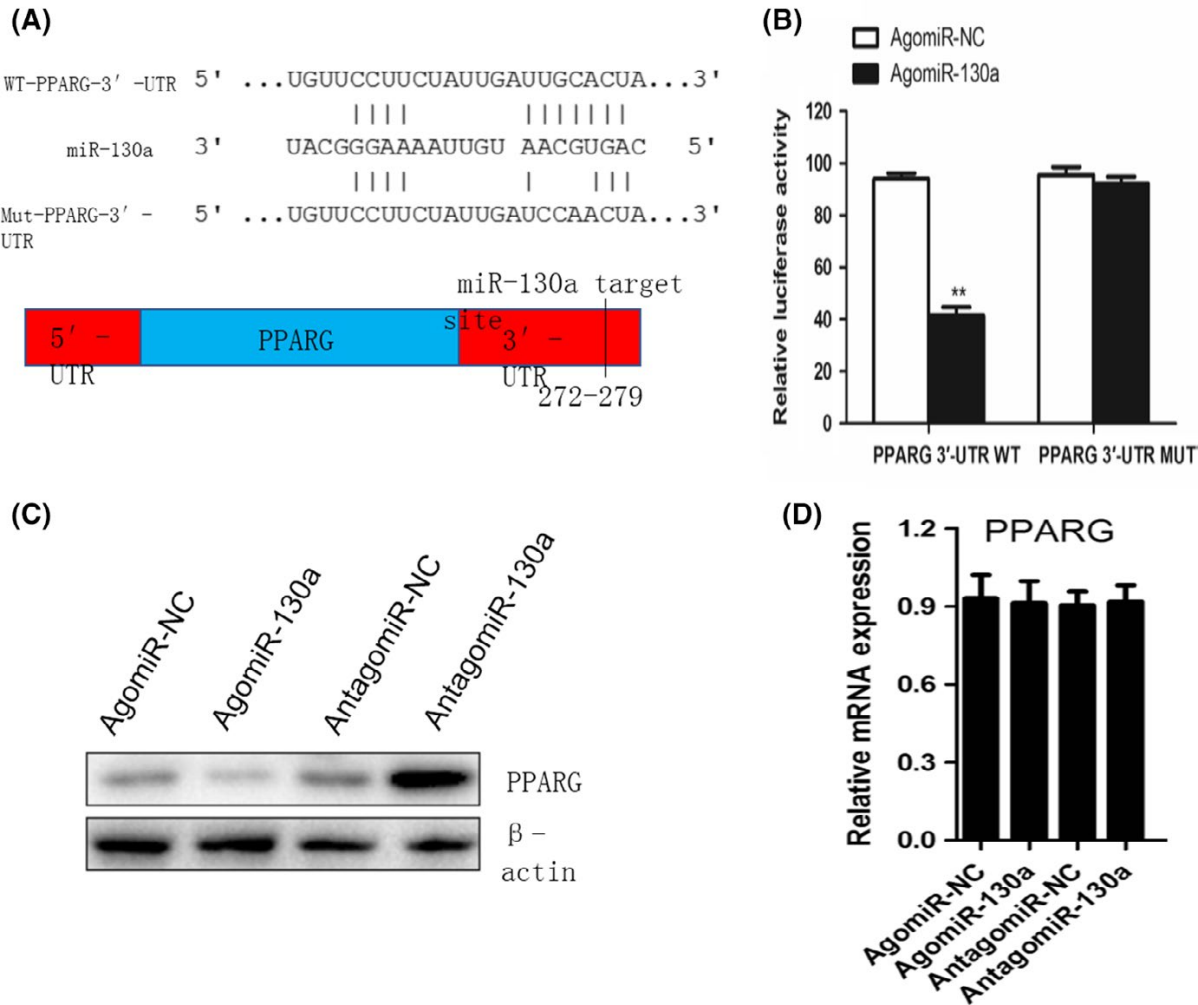
MiR‐130a targets the 3′UTR of PPARγ mRNA to regulate adipogenic differentiation. A, Schematic of miR‐130a putative target sites in mouse PPARG 3′‐UTR. CDS, coding sequence. B, BMSC were transfected with luciferase reporter carrying WT or MUT 3′‐UTR of the PPARG gene (PPARG‐3′‐UTR WT and PPARG‐3′‐UTR MUT). Effects of miR‐130a on the reporter constructs were determined at 48 hours after transfection. Firefly luciferase values, normalized for renilla luciferase, are presented. n = 3 per group. C, Western blot analysis of the relative levels of PPARγ protein expression in BMSC transfected with agomiR‐130a and antagomiR‐130a. β‐Actin was used as loading control. Data are representative of 3 independent experiments. D, qRT‐PCR analysis of levels of PPARγ mRNA expression in BMSC transfected with agomiR‐130a and antagomiR‐130a. n = 3 per group. Data shown as mean ± SD. **P < .01 vs. agomiR‐NC or agomiR‐130a (Student's *t* test)

PPARγ, important regulator of adipocyte differentiation and metabolism, is required for adipogenesis, insulin sensitivity regulation and adipocyte survival and function.^40^, ^41^ By sequence analysis, MiR‐130a had potential binding sites in the 3'UTR of PPARγ both in humans and rat (Figure 6A). Luciferase report vectors were generated with wild type and mutated PPARγ 3'UTR (WT‐pGL3‐PPARγ and MUT‐pGL3‐PPARγ). MiR‐130a inhibited luciferase activity of PPARγ, yet MUT‐pGL3‐PPARγ improved this function (Figure 6B). agomiR‐130a transfection inhibited PPARγ expression, yet antagomiR‐130a transfection induced it (Figure 6C). PPARγ mRNA had no significant differences (Figure 6D). Such results support objection of miR‐130a regulate adipogenic differentiation of BMSC by post‐transcriptional regulation of PPARγ.

## 4 DISCUSSION

With age, BMSC tend to differentiate into adipocytes but not osteoblasts, which causes osteoblast number decreased; moreover, adipocytes number increased, eventually promote the formation of osteoporosis.^8^, ^9^ According to the result we acquired, we demonstrated that miR‐130a is involved in regulating osteogenic or adipogenic commitment during the process of ageing. MiR‐130a directly targets 3′UTR of Smurf2 mRNA to inhibit expression of Smurf2 which promotes osteogenesis of BMSC. Furthermore, miR‐130a inhibits the expression of PPARγ mRNA by post‐transcriptional gene silencing to decrease adipogenesis of BMSC. Overexpression of miR‐130a in aged mice reversed BMSC differentiation from adipocytes to osteoblasts. Our results manifest that miR‐130a regulates alteration and lineage fate in BMSC at the process of ageing and plays a key role in age‐related bone loss.

It has been reported that several miRNAs play critical roles in regulating BMSC differentiation.^6^, ^7^ In our previous study, miR‐188 was found to organize osteoblast and adipocyte differentiation.^14^ However, the effect of miRNAs at ageing process between osteogenesis and adipogenesis still remains unclear. In this study, we demonstrated decreased expression of miR‐130a in elderly samples. In addition, elevation of miR‐130a results in increasing of osteoblastic differentiation and decreasing of adipogenesis in BMSC. However, inhibiting miR‐130a with antagomiR‐130a promotes adipogenic differentiation of BMSC. These results indicate that during ageing, miR‐130a regulates the BMSC differentiation directions and induces bone loss related to age.

A study has been undertaken that miR‐130a affects the process of human preadipocyte differentiate into adipocytes.^41^ In the recent study, MiR‐130a is believed to promote osteogenesis in human MSCs by targeting the PPARγ.^42^ However, no report has been published on the function of miR‐130a in regulating differentiation switch fate of BMSC during ageing. In this study, we demonstrated that during ageing, miR‐130a regulates BMSC differentiation in bone. MiRNAs combined with target mRNA on sites of the 3′‐UTR to regulate the expression of target mRNA in post‐transcriptional manner. It is well known that Smurf2 can suppress the expression of Runx2 activity,^43^ a critical osteoblast‐specific transcription factor, which leads to decreasing in osteoblast formation.^39^, ^40^ Several studies have clearly demonstrated that PPARγ is highly expressed in early stage of adipogenesis and plays a key role in adipocyte formation.^44^, ^45^ We illustrated that miR‐130a promotes osteogenesis of BMSC by eliminating the effect of Smurf2 and acts as a negative regulator of adipogenesis of BMSC by directly targeting PPARγ. These results indicated that miR‐130a post‐transcriptionally regulates Smurf2 and PPARγ expression at osteogenic and adipogenic differentiation process.

Our results demonstrated that during ageing, the osteoblast and adipocyte differentiation switches are regulated by miR‐130a through targeting Smurf2 and PPARγ mRNAs. miR‐130a expression decreased significantly during ageing. MiR‐130a depresses expression of Smurf2 to activate Runx2 expression which results in osteogenesis. Meanwhile, miR‐130a down‐regulates PPARγ expression and inhibits adipogenesis. Taken together, decreased expression of miR‐130 during ageing leads BMSC favour differentiation into adipocytes, resulting in age‐related bone loss. Thus, this study set up a novel mechanism that during ageing, miR‐130a regulates differentiation switch fate of BMSC.

As the population continues to age, bone loss has occurred with high incidence worldwide.^46^, ^47^, ^48^ A approach for improving this condition is to discover medicine which can decrease fat accumulation and increase bone formation.^49^ In this study, we identified a novel prospect for treatment. We used agomiR‐130a to treat aged mice and found the trabecular bone volume and number, as well as cortical thickness increase significantly while bone marrow fat accumulation decreases. But for the moment, most of agents available used to treat osteoporosis in the clinic are mainly focus on bone resorption but not bone formation.^50^, ^51^ This study indicates that overexpression of miR‐130a in BMSC might provide a new method for treating osteoporosis related to age.

Together, our study shows that miR‐130a is critical for regulating BMSC differentiation swift fate during ageing. These fundings supply a new target and possible mechanism for clinical therapy of age‐related bone loss.

## CONFLICT OF INTEREST

We declared that no conflict of interest exists.

## Data Availability

The data that support the findings of this study are available on request from the corresponding author. The data are not publicly available due to privacy or ethical restrictions.
